# Supplementary material for: Multiphoton In Vivo Microscopy of Embryonic Thrombopoiesis Reveals the Generation of Platelets through Budding
Source: Cells. 2023 Oct 6;12(19):2411. doi: 10.3390/cells12192411 (PMC10572188; doi:10.3390/cells12192411)
Supplement: Supplementary file 1 [file cells-12-02411-s001.zip › cells-2552323 Supplemental Figures.pdf]

Supplemental Figure S1

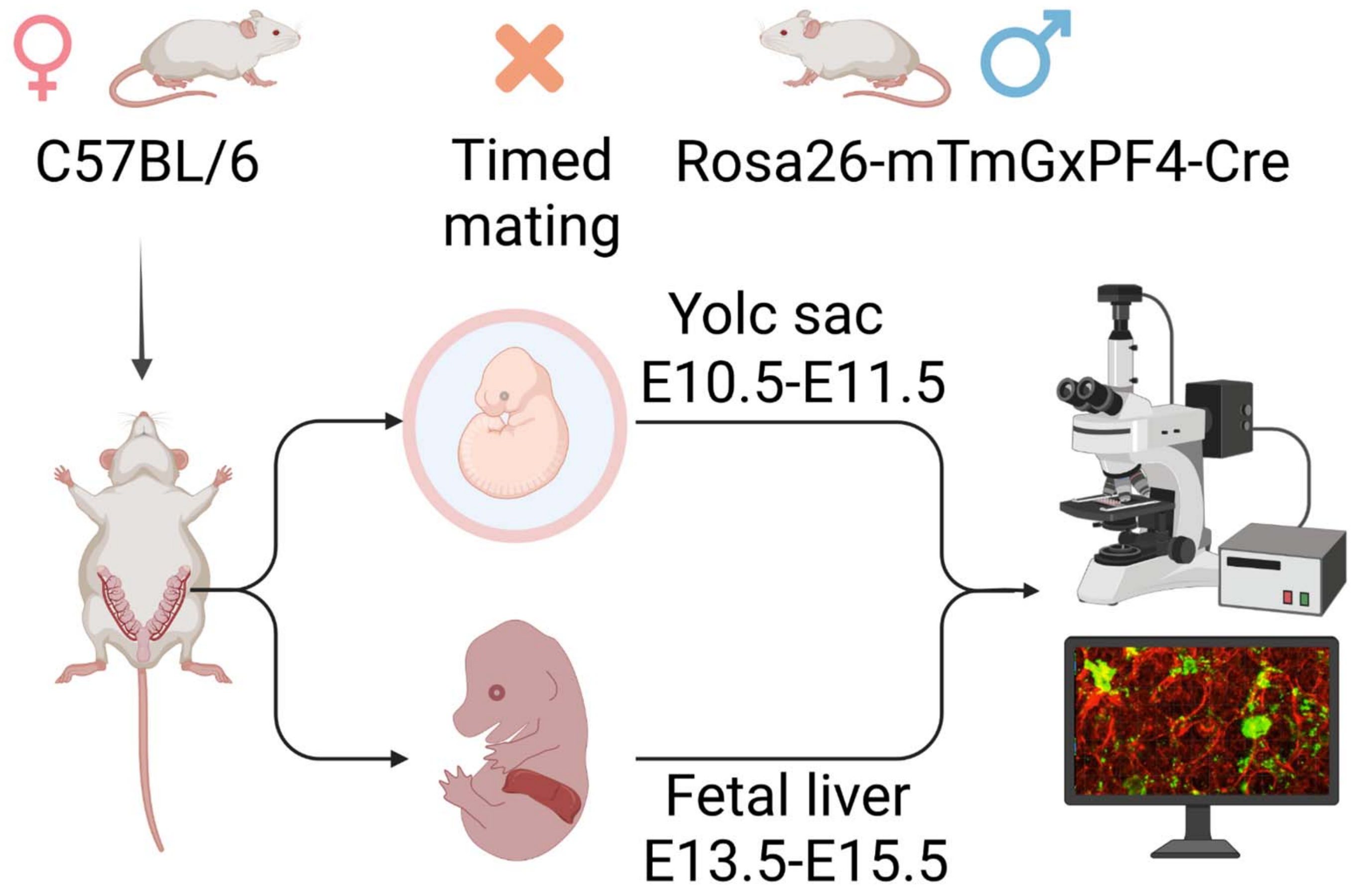

Supplemental Figure S1. Scheme of mice mating and experimental setup for yolk sac and fetal liver live imaging. The mating was done between male Rosa 26 mTmG x Pf4 Cre mice and female C57BL/6 mice. The yolk sac MP-IVM would performed at embryo day 10.5 to 11.5. The fetal liver MP-IVM would performed at embryo day 13.5 to 15.5.

Supplemental Figure S2

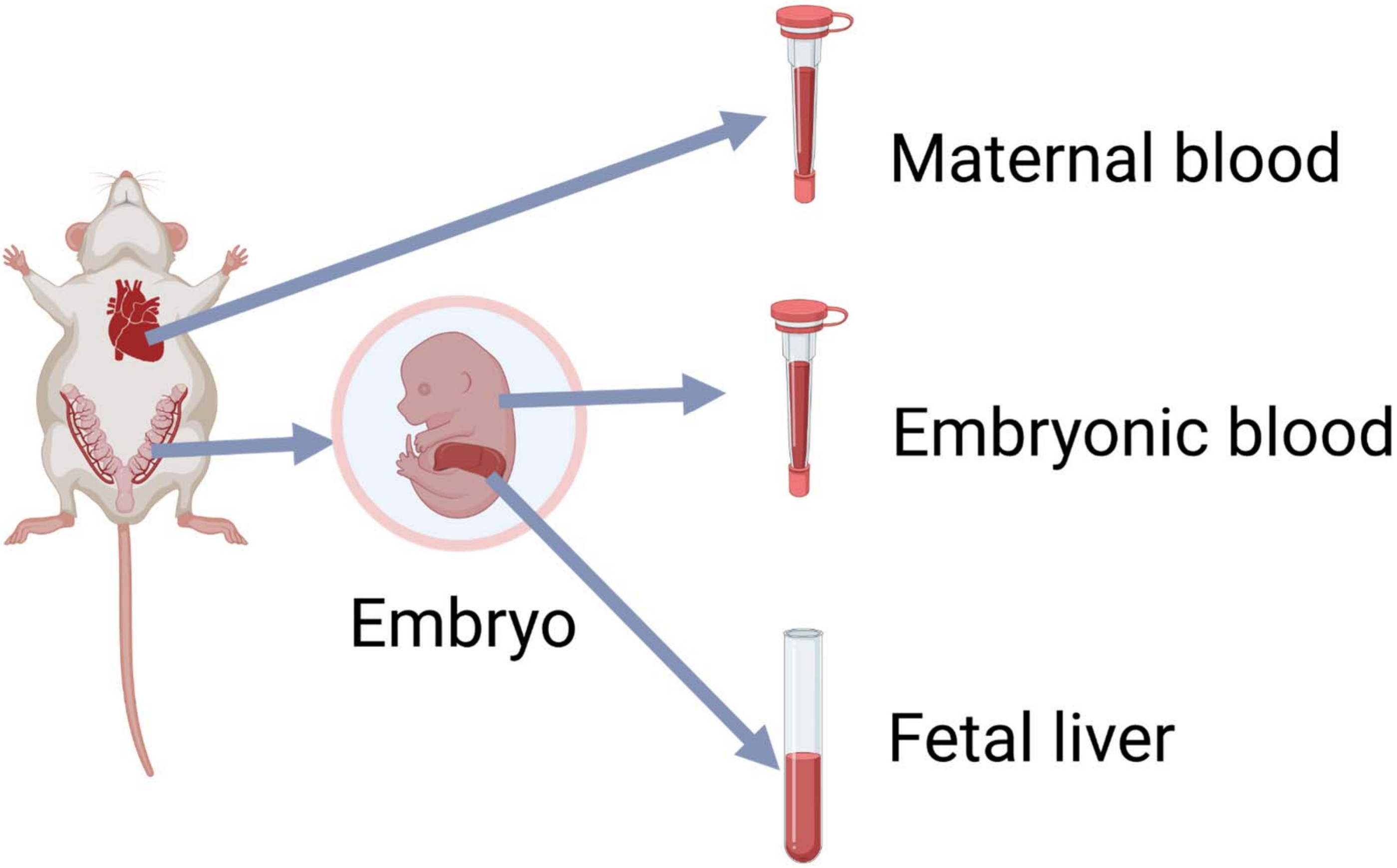

Supplemental Figure S2. Schematic presentation of mother animal and embryo processing. Full blood of maternal and embryonic blood was taken to test platelet and reticulated platelet level. Fetal liver was isolated to check the megakaryocytes.

Supplemental Figure S3

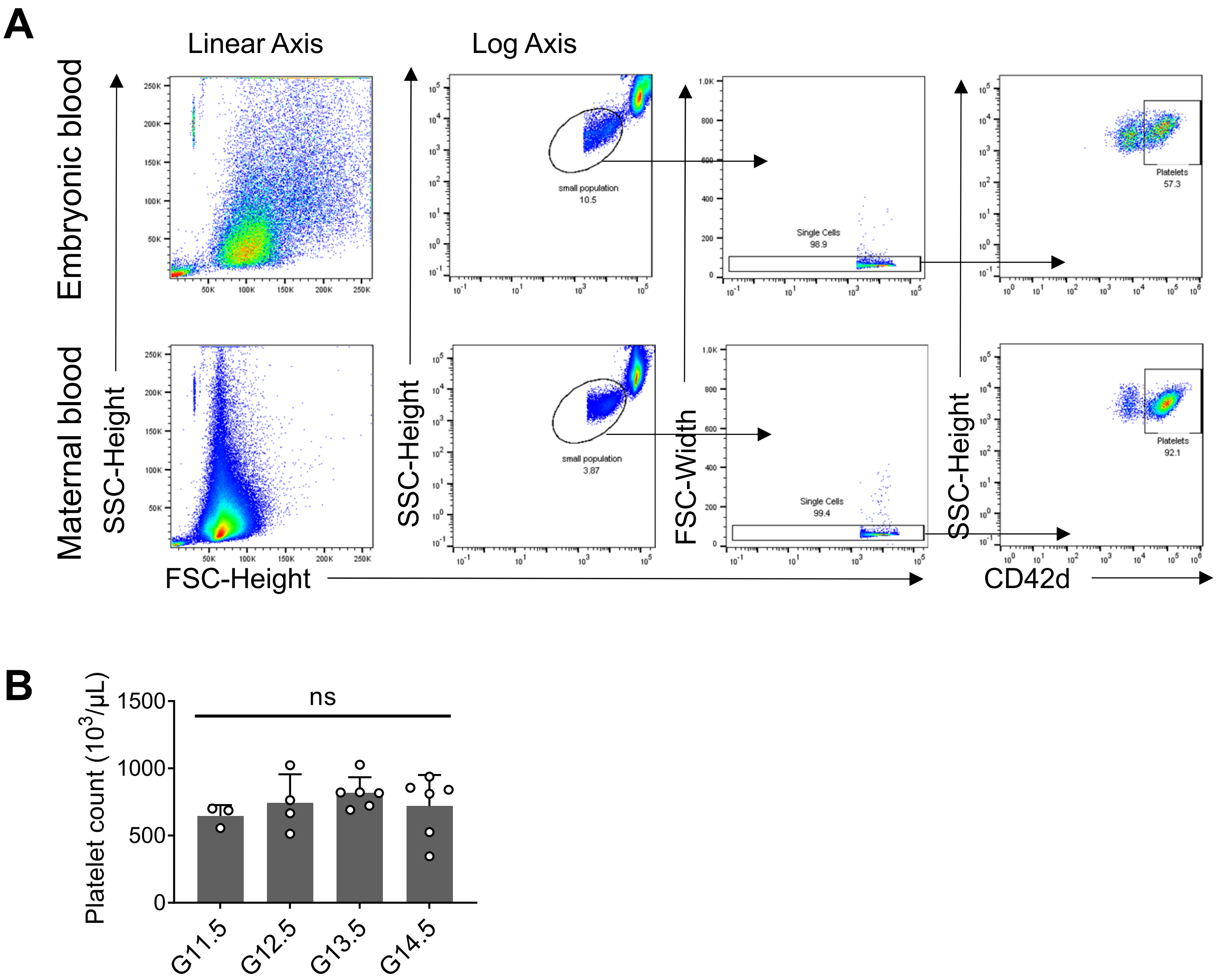

Supplemental Figure S3. Platelet flow cytometry analysis in embryonic and maternal blood. (A) Representative embryonic and maternal blood flow cytometry plots to show gating strategy of platelet. (B) platelet count in maternal blood.

Supplemental Figure S4

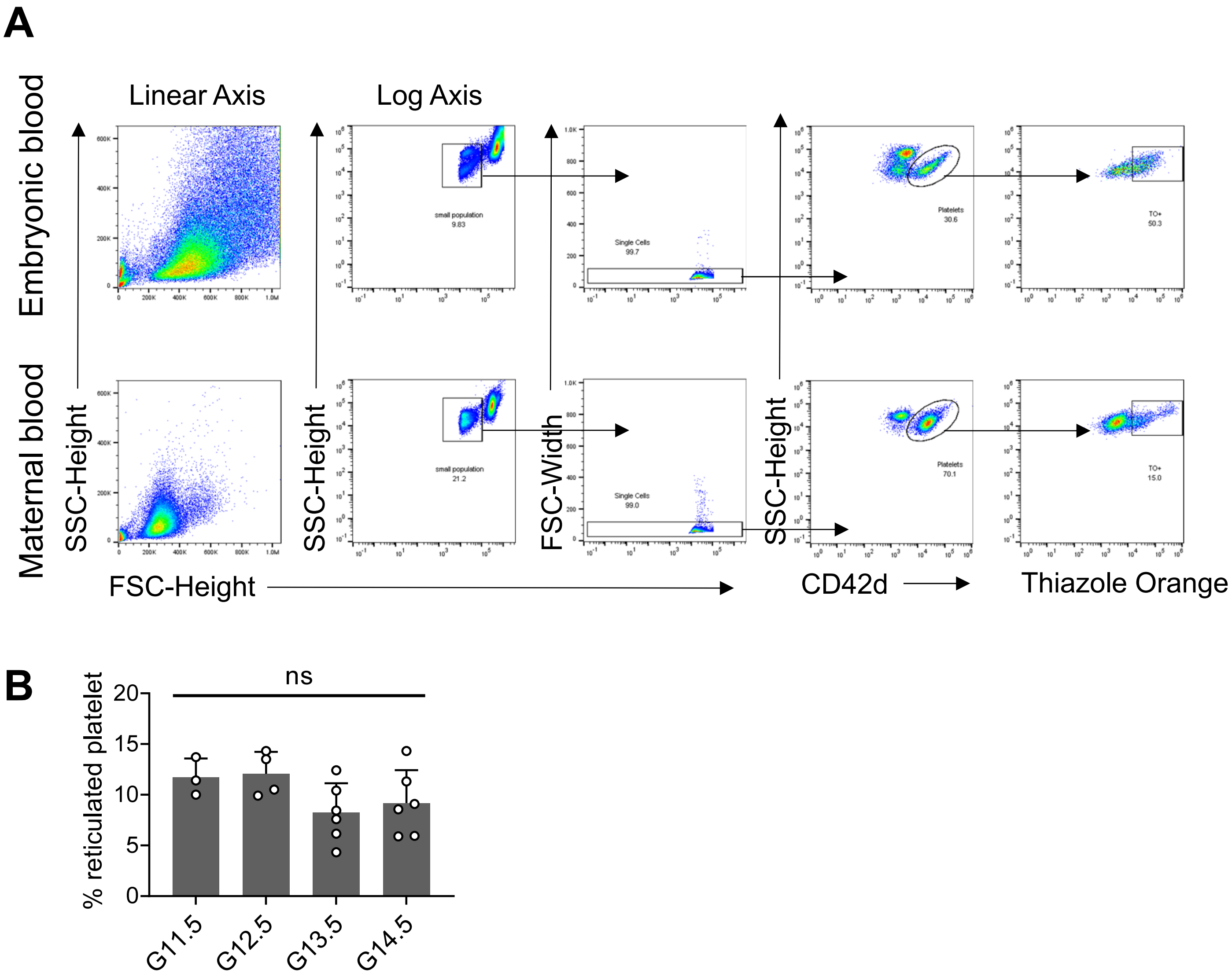

Supplemental Figure S4. Reticulated platelet flow cytometry analysis in embryonic and maternal blood. (A) Representative embryonic and maternal blood flow cytometry plots to show gating strategy of reticulated platelet. (B) Reticulated platelet count in maternal blood.

Supplemental Figure S5

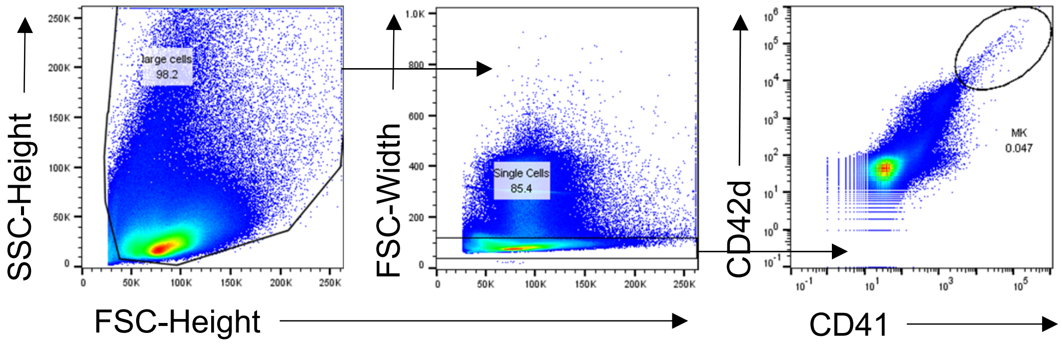

Supplemental Figure S5. Representative fetal liver flow cytometry plots to show gating strategy of MKs
